# MELD score as a predictor of mortality, length of hospital stay, and disease burden: A single-center retrospective study in 39,323 inpatients

**DOI:** 10.1097/MD.0000000000007155

**Published:** 2017-06-16

**Authors:** Jan A. Roth, Carl Chrobak, Sabine Schädelin, Balthasar L. Hug

**Affiliations:** a Division of Infectious Diseases & Hospital Epidemiology; b University of Basel; c Department of Internal Medicine; d Clinical Trial Unit, University Hospital Basel, Basel; e Department of Internal Medicine, Kantonsspital Luzern, Lucerne, Switzerland.

**Keywords:** clinical decision support system, length of hospital stay, model for end-stage liver disease, morbidity, mortality

## Abstract

The laboratory-based model for end-stage liver disease (MELD) score reflects the function of the kidney, liver, and extrinsic coagulation pathway and might be used as a general prognostic tool for the assessment of patients. We therefore aimed to investigate a potential association of the MELD score with mortality, length of hospital stay (LOS), and disease burden in a general patient population.

We performed a retrospective observational study at a tertiary referral center. From January 2012 through December 2013, all consecutive inpatients aged 18 years were eligible for the study; patients with missing MELD parameters on hospital admission and/or treatments influencing the international normalized ratio, that is, novel oral anticoagulants and vitamin K antagonists, were excluded. The MELD score on hospital admission was calculated retrospectively. The primary outcome measure was in-hospital all-cause mortality; secondary outcome measures were LOS and the number of comorbidities.

A total of 39,323 inpatients were included in the final analysis. On admission, MELD scores of 15 to 19, 20 to 29, and ≥30 points (reference <15 points) showed increased hazard ratios (HRs) for in-hospital mortality in uni- and multivariable analysis with an adjusted HR of 2.52 (95% confidence interval [CI], 1.81–3.49; *P* < .001), 2.70 (95% CI, 1.89–3.84; *P* < .001), and 8.00 (95% CI, 3.91–16.39; *P* < .001), respectively. Increased MELD scores of 15 to 19, 20 to 29, and ≥30 points were positively associated with LOS and the number of comorbidities in uni- and multivariable analysis.

In our study population consisting of adult inpatients, the MELD score on hospital admission was significantly associated with mortality, LOS, and the number of comorbidities. We suggest to prospectively validate the MELD score in inpatients as part of clinical decision support systems.

## Abstract 2

Supplemental Digital Content is available in the text

## 1 Introduction

Clinical decision support systems have been shown to improve the quality of patient care and to reduce health care costs; however, little is known about their overall impact on patient outcomes.^[1–3]^ The laboratory-based model for end-stage liver disease (MELD) score reflects the function of the kidney, liver, and extrinsic coagulation pathway and might be used as a general prognostic tool for the assessment of patients.^[4,5]^

The well-established MELD score depends on 3 readily available laboratory variables, that is, serum creatinine, serum bilirubin, and the international normalized ratio (INR).^[4,6]^ It has been developed and validated to predict mortality in patients with portal hypertension undergoing placement of transjugular intrahepatic portosystemic shunts.^[7]^ Subsequently, the MELD score was thoroughly validated in patients with a broad spectrum of liver diseases showing an excellent discriminatory power for prediction of short-term mortality.^[4,6,8]^ Interestingly, the etiology of liver disease was not found to be a relevant predictor of mortality.^[9]^

Today, the MELD score is primarily being used to allocate organs for liver transplantation, but recent studies have indicated that the MELD score might be used as a general prognostic tool in patients, independent of the presence of liver disease.^[5,10,11]^ We therefore aimed to investigate a potential association of the MELD score with mortality, length of hospital stay (LOS), and disease burden in a general patient population.

## 2 Methods

### 2.1 Study design and setting

This was a retrospective observational study performed at the University Hospital Basel, an 800-bed tertiary referral center in Northwestern Switzerland with >35,000 hospitalizations per year; treatment modalities cover all surgical and medical disciplines including kidney and bone marrow transplantations. The Ethics Committee of Northwestern and Central Switzerland approved this study with a waiver of informed consent.

### 2.2 Patient selection

From January 1, 2012 to December 31, 2013, all consecutive patients hospitalized ≥24 hours were eligible for the study. Patients younger than 18 years, with missing MELD parameters on hospital admission, and/or treatments significantly influencing the INR, that is, novel oral anticoagulants and vitamin K antagonists, were excluded from the final analysis.

### 2.3 Primary and secondary outcome

The primary outcome measure was in-hospital all-cause mortality; secondary outcome measures were LOS and the number of comorbidities, defined as the total number of recorded secondary diagnoses during the index hospitalization period.

### 2.4 Data acquisition

Relevant demographic, clinical, routine laboratory, and outcome data were collected retrospectively. Clinical data were obtained from an Oracle database of the in-house clinical information system (ISMed, ProtecData AG, Boswil, Switzerland). Routine laboratory data (i.e., the MELD parameters; serum creatinine, serum bilirubin, and INR) were extracted from our laboratory information system (X/Lab, Dorner Health IT Solutions, Müllheim, Germany). Data on patient demographics, in-hospital mortality, LOS, and *International Classification of Diseases 10th revision* (*ICD-10*) codes including secondary diagnoses were retrieved from our SAP data warehouse (SAP SE, Walldorf, Germany).

For accounting and controlling purposes, *ICD-10* diagnoses were coded by professional coders according to the German modification of *ICD-10*, version 2012: for each patient, one main diagnosis and up to 28 secondary diagnoses were recorded.

### 2.5 MELD score

The MELD score was calculated retrospectively according to the United Network for Organ Sharing modifications of the MELD formula using the first available routine laboratory data set within 48 hours after hospital admission: MELD = 11.2 × log_e_(INR) + 3.78 × log_e_(serum bilirubin [mg/dL]) + 9.57 × log_e_(serum creatinine [mg/dL]) + 6.43.^[6]^

### 2.6 Statistical analysis

The primary and secondary outcome measures were stratified by the MELD score on hospital admission using the following MELD categories: scores of <15, 15 to 19, 20 to 29, and ≥30 points.

The association of the specific MELD categories with in-hospital mortality was assessed by means of a uni- and multivariable Cox proportional-hazards regression model. The model was tested for interactions in an exploratory manner and model assumptions were controlled using residual-based methods. A uni- and multivariable quasi-Poisson regression model was used to assess the relationship of the MELD categories with LOS and the number of comorbidities. For the primary and secondary outcome measures, multivariable models were adjusted for patient age (as continuous variable), sex, and the main diagnosis category during the hospitalization period according to *ICD-10*.

All statistical analyses were performed by a biostatistician (S.S.; Clinical Trial Unit, Basel, Switzerland) using the software “R” (version 3.2.1). A *P* value <.05 was considered as statistically significant.

## 3 Results

### 3.1 Patient selection

**Figure 1:**
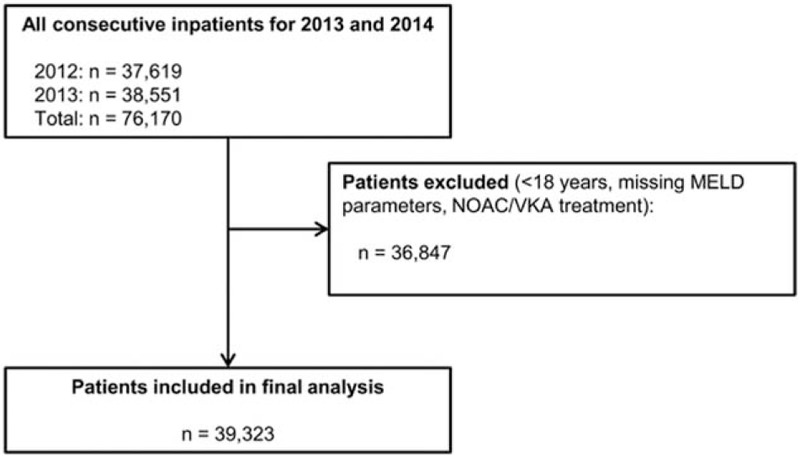
Selection of patients for study inclusion. MELD = model for end-stage liver disease, NOAC = novel oral anticoagulant, VKA = vitamin K antagonist.

In total, 39,323 of 76,170 consecutive patients were included in the final analysis after excluding 36,847 patients who fulfilled at least 1 exclusion criterion (Fig. 1).

### 3.2 Demographic, laboratory, and outcome characteristics

The median age of the study population was 64.0 years (range, 18.0–107.0 years) and 46.7% (18,376/39,323) were females (Table 1). The most frequent main diagnosis category was “diseases of the circulatory system" in 22.6% of patients (8,868/39,323) (see Table 1, Supplemental Content, which illustrates the *ICD-10* main diagnosis categories). On hospital admission, the median MELD score was 7.5 (range, 6.4–53.1) (Table 1). Overall, the in-hospital mortality was 1.0% (375/39,323), the median LOS was 5 days (range, 1–367 days), and the median number of comorbidities was 3 (range, 0–28).

**Table 1:**
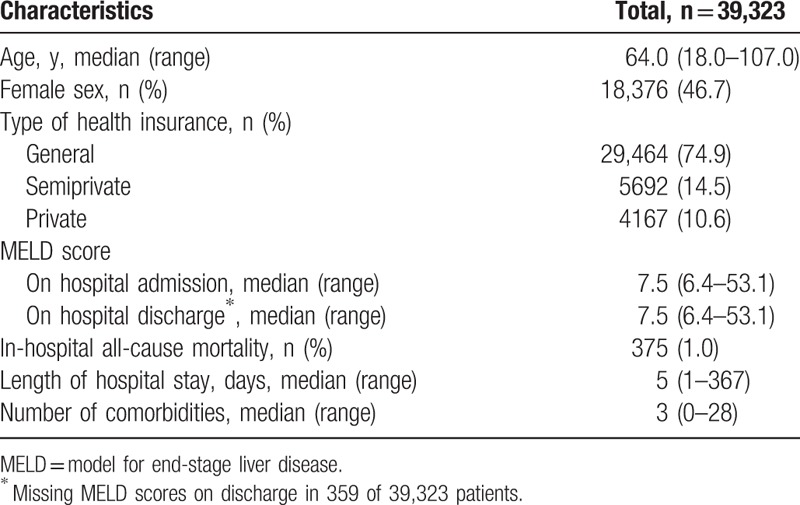
Demographic, laboratory and outcome characteristics of the 39,323 inpatients.

### 3.3 In-hospital mortality

Among the 39,323 patients, 36,488 (92.8%), 1552 (3.9%), 1214 (3.1%), and 69 (0.2%) had admission MELD scores of <15, 15 to 19, 20 to 29, and ≥30 points, respectively (Table 2). Admission MELD scores of 15 to 19, 20 to 29, and ≥30 points (reference <15 points) showed increased hazard ratios (HRs) for in-hospital mortality in uni- and multivariable analysis with corresponding adjusted HRs of 2.52 (95% confidence interval [CI], 1.81–3.49; *P* < .001), 2.70 (95% CI, 1.89–3.84; *P* < .001), and 8.00 (95% CI, 3.91–16.39; *P* < .001), respectively.

**Table 2:**
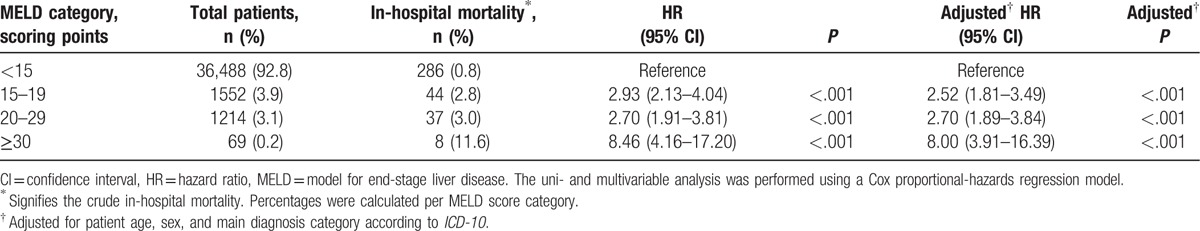
In-hospital mortality of the 39,323 inpatients stratified by the MELD score on hospital admission; uni- and multivariable analysis.

### 3.4 LOS

Overall, the LOS increased with higher admission MELD scores (Table 3). MELD scores of 15 to 19, 20 to 29, and ≥30 points (reference <15 points) were associated with increased LOS in uni- and multivariable analysis with corresponding adjusted incidence rate ratios (IRRs) of 1.23 (95% CI, 1.16–1.30; *P* < .001), 1.50 (95% CI, 1.41–1.59; *P* < .001), and 1.74 (95% CI, 1.39–2.16; *P* < .001), respectively. Increased admission MELD scores primarily identified high LOS outliers, as depicted in Table 2 of the Supplemental Content.

**Table 3:**
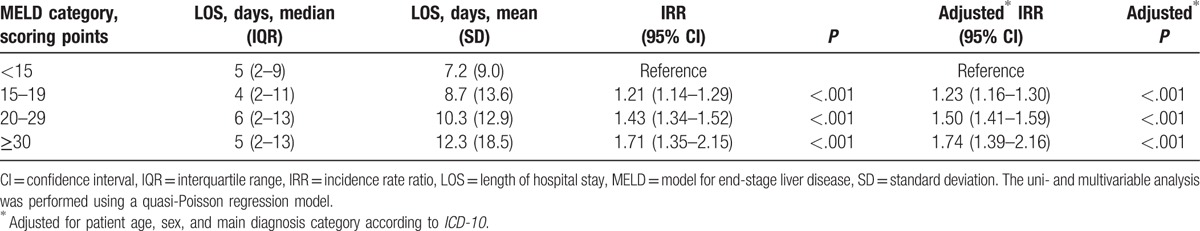
Length of hospital stay of the 39,323 inpatients stratified by the MELD score on hospital admission; uni- and multivariable analysis.

### 3.5 Number of comorbidities

Overall, the number of comorbidities increased with higher admission MELD scores (Table 4). MELD scores of 15 to 19, 20 to 29, and ≥30 points (reference <15 points) were associated with an increased number of comorbidities in uni- and multivariable analysis with corresponding adjusted IRRs of 1.46 (95% CI, 1.40–1.51; *P* < .001), 1.75 (95% CI, 1.68–1.82; *P* < .001), and 2.08 (95% CI, 1.78–2.43; *P* < .001), respectively.

**Table 4:**
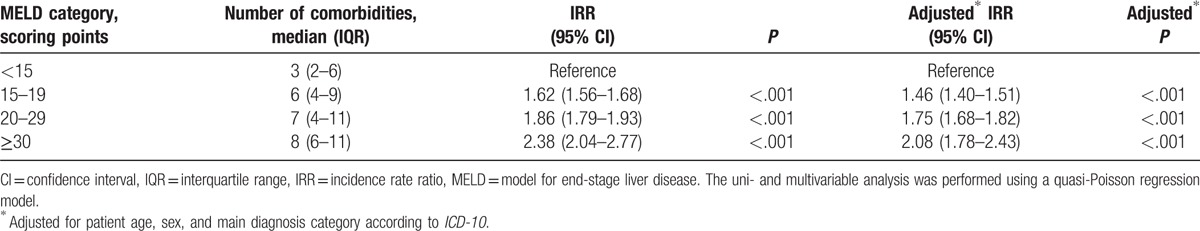
Number of comorbidities of the 39,323 inpatients stratified by the MELD score on hospital admission; uni- and multivariable analysis.

## 4 Discussion

In our study population consisting of hospitalized patients on medical and surgical wards, a higher MELD score on admission was significantly associated with an increased in-hospital mortality, LOS and number of comorbidities. The results of our study indicate that the MELD score might be used as a general screening tool to rapidly identify high-risk patients in regard to mortality, LOS, and morbidity. Till now, the MELD score has predominantly been validated in patients with liver diseases—mainly to improve the allocation process of liver transplants.^[6]^ In patients suffering from liver diseases, with or without concomitant liver cirrhosis, the MELD score has been described as a good predictor of mortality.^[4,6]^ However, the MELD score has not yet been analyzed in a general inpatient population.

The MELD score fulfills important criteria for a successful prediction model in daily clinical routine: Its three laboratory parameters (i.e., serum creatinine, serum bilirubin, and INR) are commonly and often repeatedly being measured in inpatients without depending on complex clinical variables, which are a major barrier to the application of scoring models in daily clinical routine. Furthermore, the well-established MELD parameters have been demonstrated to be significantly associated with patient outcomes in various populations such as patients with acute heart failure and septic patients.^[5,11–16]^

In our study population, the admission MELD score was not only a predictor of mortality but of LOS and the number of comorbidities as a proxy for the overall burden of disease. In regard to LOS, increased admission MELD scores primarily identified high LOS outliers (see Table 2, Supplemental Content, which illustrates the LOS quantiles of the study population). Till now, only few studies analyzed the association of the MELD score with LOS. As a surrogate marker for morbidity, Oberkofler et al^[17]^ demonstrated that increased MELD scores are an independent risk factor for a longer stay on the intensive care unit after liver transplantation. These results are in line with a recent study by Pedersen et al^[18]^ showing that the pretransplant MELD score is an independent predictor of intensive care unit LOS (adjusted odds ratio, 1.28, *P* < .001).

Of note, the median MELD score did not change during hospitalization in our cohort. It seems that MELD score changes in single patients take place over longer periods of time compared to the LOS measured in our study. This implies that the MELD score changes may be valuable for the interpretation of long-term changes in the health of a patient (>5 days in median) rather than short term.

Our study has several limitations. First, the study has been performed at a single center and 48% of patients had to be excluded from final analysis, which may have led to a selection bias and might limit the generalizability of the study results. Second, the retrospective study design allows solely to describe statistical associations, but not causal relationships. Third, in multivariable analysis, we adjusted for relevant patient characteristics, but other potentially important clinical data were not available for modeling (e.g., treatment data, presence of liver cirrhosis/chronic kidney disease). Fourth, there are studies demonstrating an interlaboratory variability in all 3 components of the MELD score with a mean interlaboratory difference of about 5 MELD points,^[19–22]^ which might further reduce the generalizability of the study results.

In conclusion, in our study population consisting of adult inpatients, the MELD score on hospital admission was significantly associated with mortality, LOS, and the number of comorbidities. We suggest to prospectively validate the MELD score in inpatients as part of clinical decision support systems.

## Acknowledgments

The authors are grateful to Prof. J.A. Schifferli, University Hospital Basel, Switzerland, for his kind support. The authors thank Dr. R. Padiyath and T. Gaida, both from the University Hospital Basel, Switzerland, for their support with the administrative patient data.

## Supplementary Material

Supplemental Digital Content
