# Supplementary material for: MELD score as a predictor of mortality, length of hospital stay, and disease burden: A single-center retrospective study in 39,323 inpatients
Source: Medicine (Baltimore). 2017 Jun 16;96(24):e7155. doi: 10.1097/MD.0000000000007155 (PMC5478332; doi:10.1097/MD.0000000000007155)
Supplement: Supplemental Digital Content [file medi-96-e7155-s001.pdf]

APPENDIX TABLE 1. Main Diagnosis Category of the 39,323 Inpatients According to the International Classification of Diseases, 10th Revision

**ICD-10 main diagnosis category[a], n (%)**	**MELD score on hospital admission**				
	**<15** (n = 36,488)	**15–19** (n = 1,552)	**20–29** (n = 1,214)	**≥30** (n = 69)	**All** (n = 39,323)
‘Diseases of the circulatory system’ (I00–I99)	7,835 (21.5)	608 (39.2)	400 (32.9)	25 (36.2)	8,868 (22.6)
‘Injury, poisoning and certain other consequences of external causes’ (S00–T98)	4,611 (12.6)	72 (4.6)	95 (7.8)	6 (8.7)	4,784 (12.2)
‘Diseases of the musculoskeletal system and connective tissue’ (M00–M99)	2,779 (7.6)	29 (1.9)	23 (1.9)	0 (.0)	2,831 (7.2)
‘Diseases of the digestive system’ (K00–K93)	2,378 (6.5)	151 (9.7)	103 (8.5)	11 (15.9)	2,643 (6.7)
‘Diseases of the respiratory system’ (J00–J99)	2,292 (6.3)	57 (3.7)	29 (2.4)	1 (1.4)	2,379 (6.0)
‘Diseases of the genitourinary system’ (N00–N99)	1,862 (5.1)	97 (6.3)	224 (18.5)	8 (11.6)	2,191 (5.6)
‘Diseases of the nervous system’ (G00–G99)	1,904 (5.2)	32 (2.1)	6 (.5)	0 (.0)	1,942 (4.9)
‘Certain infectious and parasitic diseases’ (A00–B99)	1,477 (4.0)	134 (8.6)	116 (9.6)	9 (13.0)	1,736 (4.4)
‘Symptoms, signs and abnormal clinical and laboratory findings, not elsewhere classified’ (R00–R99)	1,425 (3.9)	42 (2.7)	23 (1.9)	2 (2.9)	1,492 (3.8)
‘Pregnancy, childbirth and the puerperium’ (O00–O99)	1,118 (3.1)	1 (.1)	0 (.0)	0 (.0)	1,119 (2.8)

ICD-10 = International Classification of Diseases (10th revision), MELD = model for end-stage liver disease.

[a] The following ICD-10 diagnosis categories are not shown because the overall proportion is less than two percent: ‚neoplasms‘ (C00–D48), ‘endocrine, nutritional and metabolic diseases’ (E00–E90), ‘diseases of the skin and subcutaneous tissue’ (L00–L99), ‘mental, behavioral and neurodevelopmental disorders’ (F00–F99), ‘diseases of the ear and mastoid process’ (H60–H95), ‘diseases of the eye and adnexa’ (H00–H59), ‘factors influencing health status and contact with health services’ (Z00–Z99), ‘diseases of the blood and blood-forming organs and certain disorders involving the immune mechanism’ (D50–D90), and ‘congenital malformations, deformations and chromosomal abnormalities’(Q00–Q99).

APPENDIX TABLE 2. Length of Hospital Stay Quantiles of the 39,323 Inpatients Stratified by the MELD Score on Hospital Admission

MELD category, scoring points	Length of hospital stay [days]							
	Q 30%	Q 40%	Q 50%	Q 60%	Q 70%	Q 80%	Q90%	Q 95%
<15	2	3	5	6	8	11	16	22
15–19	2	2	4	6	9	13	21	29
20–29	2	4	6	9	12	16	24	33
≥30	2	3	5	7	12	19	40	54

MELD = model for end-stage liver disease, Q = quantile.
